# The Well-Being of Healthcare Workers During the COVID-19 Pandemic: A Narrative Review

**DOI:** 10.7759/cureus.25065

**Published:** 2022-05-17

**Authors:** Hisham Mushtaq, Shuchita Singh, Mikael Mir, Aysun Tekin, Romil Singh, John Lundeen, Karl VanDevender, Taru Dutt, Syed Anjum Khan, Salim Surani, Rahul Kashyap

**Affiliations:** 1 Critical Care Medicine, Mayo Clinic Health System, Mankato, USA; 2 Obstetrics and Gynecology, Shanti Hospital, Agra, IND; 3 Internal Medicine, University of Minnesota School of Medicine, Minneapolis, USA; 4 Critical Care Medicine, Mayo Clinic, Rochester, USA; 5 Critical Care Medicine, Allegheny Health Network, Pittsburgh, USA; 6 Psychiatry, TriStar Centennial Medical Center, TriStar Division, HCA Healthcare, Nashville, USA; 7 Internal Medicine, Frist Clinic, TriStar Centennial Medical Center, HCA Healthcare, Nashville, USA; 8 Psychiatry, Hennepin County Medical Center, Minneapolis, USA; 9 Anesthesiology, Mayo Clinic, Rochester, USA; 10 Medicine, Texas A&M University, College Station, USA; 11 Critical Care Medicine, TriStar Centennial Medical Center, TriStar Division, HCA Healthcare, Nashville, USA

**Keywords:** post-traumatic stress disorder, anxiety, depression, wellness, well-being, covid-19, healthcare-worker, sars-cov-2

## Abstract

The coronavirus disease 2019 (COVID-19) pandemic has turned into a global healthcare challenge, causing significant morbidity and mortality.Healthcare workers (HCWs) who are on the frontline of the COVID-19 outbreak response face an increased risk of contracting the disease. Some common challenges encountered by HCWs include exposure to the pathogen, psychological distress, and long working hours. In addition, HCWs may be more prone to develop mental health issues such as anxiety, depression, suicidal thoughts, post-traumatic stress disorder (PTSD), sleep disorders, and drug addictions compared to the general population. These issues arise from increased job stress, fear of spreading the disease to loved ones, and potential discrimination or stigma associated with the disease. This study aims to review the current literature to explore the effects of COVID-19 on healthcare providers' physical and mental well-being and suggest interventional strategies to combat these issues. To that end, we performed a literature search on Google Scholar and PubMed databases using combinations of the following keywords and synonyms: "SARS-CoV-2", "Healthcare-worker", "COVID-19", "Well-being", "Wellness", "Depression", "Anxiety", and "PTSD."

## Introduction and background

As of May 5, 2022, more than 510 million confirmed cases of coronavirus disease 2019 (COVID-19) have been reported worldwide, including more than 6.25 million fatalities [1]. Severe acute respiratory syndrome coronavirus 2 (SARS-CoV-2), which causes COVID-19, emerged when unexplained pneumonia cases were reported in the city of Wuhan, China [2]. On December 31, 2019, China reported an outbreak of pneumonia connected to the Huanan Seafood Wholesale market in Wuhan, Hubei Province. China's health officials confirmed on January 7, 2020, that a novel coronavirus, 2019-nCoV, was the cause of the outbreak [2]. Shortly thereafter, it was confirmed that COVID-19 had spread to several countries, including the United States, Iran, Italy, Germany, France, and Spain [2,3]. As more and more cases of the infection were confirmed, and necessary care began to be rendered at healthcare facilities, healthcare workers (HCWs) emerged as a particularly vulnerable group for acquiring this infection. In a group of 138 patients treated at a Wuhan hospital, 40 patients were HCWs. Of the affected HCWs, 77.5% worked in general wards, 17.5% worked in emergency departments, and 5% served in intensive care units. A patient infected with the SARS-CoV-2 virus who was admitted primarily for abdominal symptoms was found to be the source of infection transmission to 10 HCWs [4].

China’s National Health Commission reported that Over 3,300 HCWs had been infected with COVID-19 as of March 2020. Based on reports from the local media, 22 HCWs had already died by the end of February [5]. In Italy, it was reported that 20% of the responding HCWs had been infected with COVID-19, and some of these HCWs had died. Along with the infection risk, medical staff reported physical and mental exhaustion, the challenge of difficult triage decisions, and the devastation of losing their patients and co-workers. HCWs also expressed significant fear of spreading the virus to their families [6]. A questionnaire-based study conducted in Pakistan in May 2020 had similar findings: 94% of HCWs expressed fear of spreading the virus to their family members and friends [7]. As of May 8, 2020, Spain had reported 30,663 cumulative COVID-19 infections, which were the highest among all countries in the world at the time and accounted for 20% of the total cumulative HCW infections [8]. Italy and the Netherlands followed with 23,718 and 13,884 HCW infections, respectively. The United States was fifth on the list with 9,282 cumulative HCW infections [8].

## Review

Aim

In research conducted prior to the emergence of COVID-19, wide-ranging studies had indicated that HCWs were under severe stress due to a multitude of factors, including issues related to work-life balance, insurance and billing problems, electronic health record duties, and patient dissatisfaction [9]. At the beginning of the outbreak, HCWs were at increased risk of contracting COVID-19 due to inadequate protective measures and a lack of knowledge about the virus. Additionally, the sudden, extreme demand for protective equipment, such as gowns and N95 masks, significantly jeopardized the well-being of the HCWs. Developing standard protocols or procedures for infection prevention and control, occupational safety, and patient safety across the entire health system was necessary to ensure the safety of both HCWs and patients. Studies on severe acute respiratory syndrome (SARS) and the Middle East respiratory syndrome (MERS) have provided insights into the stresses, traumas, and psychological illnesses associated with communicable respiratory diseases and successful interventions made to combat them [10]. While these studies may provide insights relevant to the current outbreak, the COVID-19 pandemic has introduced unique challenges that require further investigations. This study was conducted to aggregate information from the current literature regarding the impact of COVID-19 on HCWs' well-being with the goal of developing practical interventions. Due to the evolving nature of the pathogen, new evidence regarding the negative impact of COVID-19 on the health of HCWs is constantly emerging.

Methods

We searched the databases PubMed and Google Scholar by using the keywords "SARS-CoV-2", "Healthcare-worker", "COVID-19", "Well-being”, “Wellness", "Depression", "Anxiety" and "PTSD" from December 2019 to March 2022. Eligible studies included in this review were articles published in English, whose primary focus was the effect of COVID-19 on the physical and mental well-being of HCWs. Studies that were excluded were articles published in languages other than English, articles related to other pandemics, and articles that studied the well-being of non-HCWs.

Results

Physical Well-Being

Between January 3-11, 2020, 30 infected HCWs with novel coronavirus were referred to the Jianghan University Hospital. There were 10 men and 20 women, including 22 physicians and eight nurses, aged 21 to 59 years. All these HCWs had come into close contact (within 1 meter) with a patient infected with COVID-19, and the 30 patients were classified into 26 mild or moderate cases and four severe cases. This study concluded that HCWs are at higher risk of contracting COVID-19 and that the risk of infection increases with prolonged contact times with infected patients [11]. A retrospective cohort study further analyzed the risks associated with COVID-19 infection among HCWs. They examined 72 frontline HCWs aged 21-66 years and concluded that working in Pulmonology and Infectious Disease departments was associated with an elevated risk of contracting the infection [12]. This study also found that exposure to patients without proper personal protective equipment (PPE), long hours of daily contact (≥15 hours), close patient contact (12 times/day), inadequate hand hygiene, and diagnosis of COVID-19 in a family member were associated with an elevated risk of contracting COVID-19. The prevalence of COVID-19 viral respiratory illnesses in HCWs was reported at 1.6-44% [12]. The most commonly reported symptoms were fever, cough, fatigue, and myalgia. Other symptoms were headache, chest symptoms, dyspnea, diarrhea, nausea/vomiting, and hemoptysis [12]. A study from Germany reported that 0.33% of healthcare staff acquired symptomatic disease [13]. The SEMI-COVID-19 registry in Spain analyzed a cohort of 4,393 patients, out of which 419 were HCWs. It revealed a 9% hospital admission rate for HCWs. Sepsis (3.9% versus 1.7%) and in-hospital heath (4.8% versus 0.7%) were lower in HCWs compared to the general population, but other complications such as pneumonia, thromboembolism, and ICU admission showed no difference [14]. Professional exposure did not seem to increase the severity of the disease. The severity was attributed to known risk factors and comorbidities.

There is a unanimous consensus that the use of N95 decreases the chances of contracting viral respiratory illnesses [15]. Studies suggest that better protection is obtained with coveralls and long gowns, but it made donning and doffing difficult, leading to low user satisfaction and overall greater contamination [16]. Prolonged use of N95 and PPE kits has reportedly led to device-related pressure injuries, which can be combated with foam and hydrocolloid dressing [17]. Polyurethane foam-lined respirators have reduced injuries from 84.7% to 11.1%. They also improved pain scores and redistribution of pressure across the face [18]. Protection with barrier boxes and an air-purifying respirator with a hood has helped in decreasing the transmission during intubation [19]. In low-resource settings or in cases of shortages, a well-washed cloth mask can be effective [20]. Handwashing adherence can be improved by the health action process approach (HAPA) [21]. Video-based instruction in PPE donning and doffing for medical students and junior doctors provides fast and resource-efficient training [22,23].

Dermatological Manifestations

Dermatological manifestations have been widely reported among COVID-19 patients [24]. The most common dermatologic problems experienced by HCWs involved in treating patients with COVID-19 were associated with the use of PPE. The nasal bridge was most commonly affected, followed by hands, forehead, and cheeks. The most common symptoms included desquamation and tightness [25]. The risk of skin damage in the corresponding site was greater for medical personnel who had worn their devices for six hours or longer. However, wearing a face shield for a long time was not a significant risk factor for skin manifestations over the forehead [25]. More frequent hand hygiene (>10 times per day) may be associated with increased risks of hand skin damage compared to longer glove-wearing time [25]. Other than allergic or irritant contact dermatitis, cutaneous vasculopathy, micro thrombus-related changes, urticaria or angioedema, morbilliform/maculopapular exanthems, erythema multiforme, and vesicular eruptions have been reported at a rate of 5-20% [26].

Mental Well-Being

A. Anxiety and Depression

With the outbreak of the COVID-19 pandemic, its immense impact on the mental well-being of HCWs has become evident [27,28]. Most of the initial studies were from China; however, in the course of one year, studies from various other healthcare systems have reported comparable outcomes. Pappa et al. analyzed 13 cross-sectional studies with 33,062 participants and found a pooled prevalence of anxiety at 23.2% and depression at 22.8% [29]. Luo et al. incorporated 62 studies in their analysis and reported a prevalence of 33% (28-38%) and 28% (23-32%) of anxiety and depression, respectively [30]. The primary cause leading to this was the enormous and unanticipated workload causing physical exhaustion. Moreover, the lack of adequate personal equipment and the consequent risk of nosocomial transmission compounded the anxiety among HCWs. The need to make ethically difficult decisions like triage and life support was also a contributing factor. Sociodemographic factors like younger age and being female were associated with a higher prevalence. Individuals involved in specific occupational roles such as those working in direct care and nurses have had an increased occurrence of symptoms as compared to administrative staff [31]. Similar conclusions were also seen in the study by Al Maqbali et al., where the pooled prevalence of anxiety, depression, and insomnia in nurses was 37%, 35%, and 43%, respectively [32]. The prevalence was higher in HCWs than in the general public. This was in contrast with the SARS epidemic in 2012. The mental wellness of the public deteriorated over time, while that of HCWs improved after the peak of the epidemic, which could be due to a lack of knowledge and sociopolitical impact. The protective factors identified are social support, financial stability, and resilience [33,34].

B. Stress and Insomnia

Wu et al. have investigated the prevalence of depression, anxiety, stress, and insomnia during the COVID-19 pandemic. Stress was reported in 41.5% of HCWs, which was less than in patients with comorbidities such as cancer, diabetes, or chronic kidney disease (49.1%). Also, 47.3% of HCWs reported insomnia, which surpassed the rate observed among the general population and students including university, college, and middle school students [28,35]. This may be due to the fear of the consequences of infection or anxiety about being stigmatized or discriminated against due to COVID-19. The length of quarantine duration and fear of getting infected could be the deep-rooted factors behind stress [36]. The prevalence of acute stress disorder has been reported to be as high as 40% in HCWs during and after epidemics; however, the increased prevalence in female HCWs is not seen [37]. Fear of contamination could result in obsessive-compulsive manifestations [38].

C. Post-traumatic Stress Disorder

In a systematic review including 97,333 HCWs, two out of every 10 HCWs were noted to have PTSD [39]. PTSD was more common among quarantined physicians [39]. Another study reported a prevalence of about 30% in COVID-19-symptomatic cases in the general population and 20% in HCWs [40]. The possibility of delayed-onset PTSD (after three years) during the COVID-19 pandemic cannot be overlooked, and HCWs at high risk should be followed up in the coming years, as the outcomes observed during the earlier SARS pandemic are indicative of its prevalence [41].

D. Obsessive-Compulsive Disorder

Fear of contamination could result in obsessive-compulsive manifestations. French et al. have described a patient whose obsessive-compulsive disorder (OCD) symptoms were acutely exacerbated by the COVID-19 pandemic and the media coverage surrounding it. These symptoms resulted in significant functional limitations and included increased ritualistic handwashing and cleaning, unwillingness to leave home due to fear of spreading infection, dropping out of an educational course, having minimal or no social interaction with friends or family, and only eating canned foods since they are perceived to have a lesser contamination risk of COVID-19 [38].

E. Occupational Burnout

When comparing the frequency of burnout between HCWs in usual wards (UWs: non-COVID-19) and HCWs on the frontline (FL: COVID-19) wards, researchers have noted that the latter experienced a lower frequency of burnout and were less worried about becoming infected. According to the available data, closer proximity to decision-makers could benefit FL workers, who may feel more empowered over their situation than other HCWs. Based on the study results, it is certain that both UW staff and those working in FL wards must be kept in mind when drafting policies and procedures to support the well-being of HCWs in response to the COVID-19 crisis [42]. A striking contrast was seen in the study, which used the abbreviated Maslach Burnout Inventory (aMBI) to assess burnout and career satisfaction among neurosurgeons in the United States. They found that rates of burnout were actually lower when compared with the pre-COVID-19 era [43]. The authors of this study suggest that decreased working hours due to the pandemic may have contributed to decreased burnout and increased career satisfaction [43]. Another study from the United States examined the effect of the COVID-19 pandemic on burnout among physicians in outpatient interventional pain management practices using a survey that assessed physicians’ concerns and outlooks regarding their careers [44]. This study found that most responding physicians expressed concerns about finances, reduced staffing, and maintaining adequate PPE availability due to the pandemic. Additionally, this study found that 60% of respondents felt that the COVID-19 pandemic had a negative impact on their practice, and 52% of respondents currently felt burned out [44]. These studies suggest that the COVID-19 pandemic may have disproportionately increased burnout among outpatient providers. In healthcare, burnout harms patients, HCWs, and the healthcare system itself. With the emergence of the COVID-19 pandemic, burnout has increased to the point that it poses a threat to the proper functioning of the healthcare workforce. It is predicted that elevated burnout and other indicators of stress will persist well after the pandemic [45].

Discussion

As of May 6, 2022, there were more than one million HCW cases and more than 4,000 HCW deaths related to COVID-19 in the United States [46]. Previous epidemics have provided significant insights into what can be done to reduce the psychological distress of HCWs during a pandemic. The most important strategy is to provide HCWs with adequate PPEs and train them on using them properly. It is essential that adequate staffing be made available to clinicians to receive necessary self-care while remaining vigilant [47]. Several hospitals had already experienced severe nursing shortages before COVID-19; the situation was much worse during the first few pandemic waves. This can be addressed by practical measures such as telemedicine and postponing elective procedures to relieve some burden. In addition, authorities should focus on providing a one-day licensure approval process for nurses and other medical professionals promptly to tackle the surge [48].

Since the outbreak of SARS in 2003, direct-care providers have reported high rates of recurrent PTSD, even years after the crisis had ended. Therefore, short-term and long-term mental health services need to be made available to all healthcare professionals [47,49]. Research has shown that active coping, acceptance, cognitive-behavioral skill-building, deep breathing, stress reduction strategies, mindfulness, gratitude, health coaching, and positive framing can help achieve better mental health [47]. The MINDBODYSTRONG integrated mental health and physical health skill-building program that combines cognitive and behavioral strategies targeting mental and physical well-being has proven beneficial [50]. Trials are ongoing to analyze the beneficial effects of biweekly psychotherapy sessions on burnout using the Death Café model, which includes informal discussions focusing on death, loss, grief, and illness [51].

The Med-Stress internet intervention study included self-efficacy and social support enhancement modules to improve outcomes in HCWs. Job stress decreased with self-efficacy exercises, and burnout was alleviated with additional therapy of mindfulness, relaxation, and lifestyle and cognitive restructuring [52]. We must eliminate stigma and raise awareness about and screen for depression and PTSD in hospitals and HCWs and implement systems to handle them effectively. In light of the ongoing COVID-19 crisis, we must include encrypted screening tools for suicidal ideation and depression and evidence-based interventions [53]. It is critical to maintain unit cohesion during times of crisis so that social support can be provided, help can be found effectively, and the stigma associated with stress can be reduced. These practices nurture coping skills, promote adaptation, and foster resilience [54].

Occupational health and safety policies and procedures should be followed effectively, including staff screening and testing, staff illness protocols, and safe return-to-work policies to allow staff to stay home if unwell, without loss of income; reports and investigations of unprotected exposures and contacts with suspected or confirmed COVID-19 cases that are blame-free - management protocols to ensure sufficient staff; appropriate shifts, safe staff-to-patient ratios, and ventilation; rest periods in areas with adequate space and reminders to staff to continue adherence to IPC procedures [55].

Strategizing interventions is necessary for improving HCWs' physical and mental well-being. These interventions can help provide necessary guidelines for organizations to protect the well-being of HCWs. These measures can also be protective in the future in times of acute need.

Based on the available literature, the following strategies appear essential for HCWs' well-being (Table 1).

**Table 1:** Summary of strategies for healthcare workers' well-being

Strategies for healthcare workers' well-being
Prioritizing occupational health and safety policies, guidelines, and procedures laid down by WHO, including (1) staff testing, (2) staff illness protocol, and (3) safe return-to-work policies
Accessible, appropriate PPE for all healthcare workers to protect themselves and their loved ones from infection
All healthcare workers should have access to short- and long-term mental health services
Put systems in place to effectively handle depression and post-traumatic stress disorder, along with evidence-based interventions, eliminating stigma, and raising awareness about and screening for them at hospitals and healthcare centers
Prioritize sleep, spend time in nature, practice mindfulness, exercise regularly, connect with your community or faith-based groups, and find ways to relax when stressed
Mental health and social support services should be made available for healthcare workers, including information on work-life balance, risk assessment, and mitigation

Limitations

Due to the rapidly evolving nature of the pandemic, updated reviews are required in the upcoming months. Many psychological difficulties experienced by workers during the initial days of the pandemic will change over time, and hence the considerations for the future well-being of workers are difficult to assess. Each study conducted on the subject involves a population that may be biased in certain ways or not fully representative of the working population. Moreover, findings in terms of study responses to stress and crisis may vary from country to country based on economic conditions, the system of healthcare assistance, and culture.

## Conclusions

Their active role in providing care for patients with COVID-19 places HCWs at immense risk of physical and mental health complications. The risks include exposure to the pathogen, psychological distress, occupational burnout, fatigue, long working hours, and stigma. Improved workplace infrastructure and effective and shared anti-contagious measures for HCWs, including regular PPE supply and provision of mental health and support services, are possible actions to ensure that HCWs do not experience adverse psychological and physical effects. It is a challenge to live and work in the age of COVID-19. Prioritizing physical and mental well-being is the first step to getting back to normalcy. We should strive to learn from this pandemic to prepare for the next pandemic to avoid preventable deaths.
